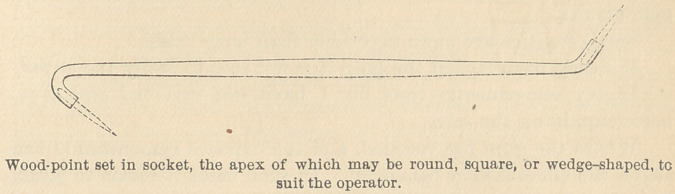# What Value Has Argenti Nitras as a Therapeutic Agent in Dentistry?

**Published:** 1891-10

**Authors:** E. A. Stebbins

**Affiliations:** Shelburne Falls, Mass.


THE
### International Dental Journal.

Vol. XII. October, 1891. No. 10.

### Original Communications.[1]

### WHAT VALUE HAS ARGENTI NITRAS AS A THERAPEUTIC AGENT IN DENTISTRY?[2]

BY DR. E. A. STEBBINS, SHELBURNE FALLS, MASS.

Mr. President,—The object of this paper, and the presentation of these patients and specimens, is to bring the subject to the notice of the profession more fully, and stimulate to further investigation, and, if found practicable, to bring it into more frequent use.

Allow me briefly to take you over some of the way I have come in my study and experiments. Every observing practitioner who has had a few years' experience has seen teeth that have begun to decay on the labial or buccal surfaces, then, from some cause, the decayed portion has taken on a very dark color, and the progress of the decay has ceased.

The same conditions have been observed in approximal cavities when the adjoining teeth have been extracted; also when, by mastication, the teeth have been worn off till approximal cavities have become exposed. In some mouths a large number of such cases may be noticed.

---

[1] The editor and publishers are not responsible for the views of authors of papers published in this department, nor for any claim to novelty, or otherwise, that may be made by them. No papers will be received for this department that have appeared in any other journal published in the country.

[2] Read before the Connecticut Valley Dental Society, June 11, 1891, and the Massachusetts State Dental Society, July 9, 1891.

In the process of change, the ordinary color of decaying tooth-substance becomes darker and darker, till, in some instances, it is nearly black. It also becomes quite hard.

These "black spots" do not decay until, by some means, the "crust" is broken, or disease approaches from an adjacent portion of the tooth.

We are all familiar with the appearance of cavities which have been filled with amalgam.

Can this black-crust condition be produced instantly, and at will? Will it remain? If so, we have a boon of great value.

The constituents of decaying tooth-substance, the elements of the remedy, and the compound formed by the union of the two, with practical results, will be evidence towards a solution of our query.

The "United States Dispensatory" says of argenti nitras: "The solution stains the skin of an indelible black color, and is itself discolored by the most minute portion of organic matter, for which it forms a delicate test. The affinity of this salt for animal matter is evinced by its forming definite compounds with albumen and fibrin. . . . When nitrate of silver, in a pure state, is brought in contact with living tissue, it acts as an escharotic. Owing to a formation of a dense film of coagulated albumen, the depth of its action is very limited; the albuminous coating is at first white, but soon becomes blackish, owing to the reduction of the silver."

The "New American Cyclopædia" says: "With albumen, fibrin, etc., it forms insoluble compounds. . . . From recent microscopical examinations very carefully made by Mr. T. J. Herapath, of England, upon some obscure marks found upon wrappers of mummies, there is reason to believe that the ancient Egyptians were acquainted with the compound of nitrate of silver."

"Taft's Operative Dentistry" says, under "Treatment of Sensitive Dentine:"

"*Nitrate of silver.*—This salt is a powerful caustic, whether applied to soft parts or bony tissue. Its action is somewhat complex. Nitric acid is liberated by the decomposition of the salt when in contact with organic matter. Nitrate of silver has a strong affinity for albumen, uniting with it without difficulty, and the compound thus formed is soluble in nitric acid. When the nitrate is applied to the skin, the immediate result is a whitish mark, caused by the union of the salt with the albumen of the cuticle; but this soon turns black by the reduction of the salt and the liberation of the oxide of silver, when for each atom of this set free there is liberated an equivalent of nitric acid. There is here, then, an agent that acts promptly on the gelatinous portion of the tooth, destroying vitality to the extent of the combination which takes place, and that, by the decomposition of part of the salt and the consequent liberation of part of the acid, also acts with energy on the calcareous portion. The compound formed by the nitrate with organic constituents of the tooth is insoluble, except with a few substances, and therefore protects the subjacent parts; and the precipitation of the reduced oxide on the surface, it is claimed, affords some additional protection.

"The insolubility of the compound above mentioned prevents an absorption of the nitrate by the dentine, and renders its action necessarily superficial. When the nitrate is neutralized by a union with it of an equivalent of the constituents of the dentine, no further chemical action is possible. The compound formed by this union is soluble in a dilution of the nitrate, and if this be applied in too great a quantity there may be a larger loss of substance than is desirable or at all necessary; for as long as free nitrate remains in solution in the cavity, the insoluble compound is not precipitated, and the surface is therefore exposed to the continued action.

"It is preferable to employ the nitrate in the solid state, or, when this is not practicable, in a concentrated solution, and small quantity, rather than in a copious dilution and repeated application.

"From the observations already made, it is quite evident that no harm can result to the tooth from a proper application of this agent, beyond the portion of it immediately acted upon. The nitrate cannot be absorbed by dentine, but it stimulates the adjacent dentine to more healthy action."

Following is a letter from Professor Charles Mayr, of Springfield, Mass., in response to my inquiry as to what the chemical effect of nitrate of silver is on decaying tooth-structure. This is not the first time Professor Mayr has contributed valuable information for this Society, and for which we hold him in high esteem.

"In regard to the subject in question I would say that many points have to be considered in regard to the effect of nitrate of silver on tooth-substance. The first is the purely chemical effect.

"Now, decay contains lactate of lime (accepting Dr. Miller's analyses, which agree perfectly with my own finding) and organic matter,—the whole permeated by ravenous microbes. The chemical action of silver on the lactates is not very rapid, but after a short lapse of time they are oxidized and the silver reduced. On the organic matter the nitrate of silver acts much more rapidly, being reduced by it, and of course in its turn destroying the organic matter, but, most of all, it acts as a powerful germicide. Silver salts are nearly as hostile to germs as mercury salts, but have the increased advantage of also destroying the products of the germs, which mercury salts do not to this extent.

"In regard to its effects upon the dentine, I should also say that the various chemical constituents of the dentine have to be considered.

"On the phosphates of lime the action is slow, but terminates in forming nitrate of lime and phosphate of silver.

"The carbonates are rapidly acted on, forming carbonate of silver and nitrate of lime.

"The decomposition in both cases is not complete. A small amount of undecomposed silver salt would remain on the spot touched by the nitrate of silver, and a small amount of undecomposed lime salts. The organic substances would slowly be destroyed by the nitrate of silver.

"Of course there would also have to be had the physiological action of the dissolved silver on the nerves abutting at the spot touched.

"It is not improbable that the silver acts by decomposing the chlorides,—potash and soda combine with the nerve-substance essential to its comfort and well-being; at the same time, it would coagulate the albumen in the nerve-substance, thus forming a plug consisting of albuminate of silver, chloride, phosphate, and carbonate of silver, which plug would be an insulator against pain, and would be equivalent to destruction of the nerve-tissue for the space acted upon."

The use of a solution of the salts has been ineffective in my hands.

For sensitive dentine or cementum I use the salts in the same way as for decay. Senile teeth from which the gum has receded and become inflamed, and the exposed cementum is sensitive on the application of a tooth-brush, are very much benefited by this treatment. The brush can be used with impunity, and the gum becomes harder and much more nearly normal.

Dr. R. R. Andrews has demonstrated very clearly that in some cases decay extends very far into the teeth when the periphery is small. If the decay reaches the pulp the caustic effect of the silver will disturb the quiet of that organ and cause pain. My experiments thus far teach me to be cautious in cases of deep decay.

Where the decayed portion is very thick, coagulation of the salts and organic matter may be so complete as to stop the advance of the union before the deepest lamina is affected.

Argenti nitras is not a panacea for every ill known to the dental profession. In some patients' mouths the visible effects will disappear entirely in a year. In other cases it will be effective in part of the cavities, while in other cavities of the same mouth it will be partially effective or not effective at all. What are the causes of these different results?

A serious objection to the use of this agent is the color it produces where there is the slightest decay. Where the tooth is not decayed no change is produced.

Some patients would object to have it applied to any tooth on account of the color. Others would be pleased to have it used where the cavity would not be seen. But there are thousands who must have some such treatment, or become edentulous. Children who are too sensitive to have teeth filled, or whose parents have not the means to pay for filling, must have some relief, or suffer untold misery, and lose their temporary teeth too soon, thereby involving themselves in life-long troubles. Having witnessed the relief of so many children from constant agony, and their exemption from toothache for years, by the application of this agent, it seems to me it is worthy a larger place in our practice.

For several years I have been testing its effects as opportunities have been presented, though not in that thorough and systematic way I would desire.

By the patients, the specimens I will exhibit, and the cases to be related, I will endeavor to present a fair representation of the subject as far as my investigations have gone.

I will give an account of five cases.

The patients present may be seen, with records of their treatment. The specimens also will be passed around attached to cards on which are the records thereof.

Case I.—F. H. M., a man about thirty years of age. September 6, 1888, I treated superficial decay in the labial surface of superior cuspids and first right bicuspid; also buccal surface of first inferior molars,—all near the gum. None of these cavities have decayed since.

This patient has twenty-eight teeth, all but six of them having from one to three fillings each. Business keeps him from attending this meeting.

Case II.—Aged about thirty-five years. Right inferior second molar had a small cavity in buccal surface treated. One year and three months after, decay had begun just under the enamel. Recauterized. One year and nine months later, found decay again aroused just under the enamel. On excavating for filling, found that the decay had not gone deeper,—only enlarged the periphery. Superficial decay on the left molar of same mouth appeared to have kept perfectly after two years and nine months.

CASE III.—The following communication is from W. H. Ashley, M.D.:

"Some twenty-odd years ago I was kicked in the mouth by a horse, cutting both lips through and splitting the aveolar process of the superior maxillary. From that time I suffered more or less trouble with my teeth. The lower ones frequently caught over the upper ones, severely wrenching them during mastication. The upper teeth were strapped outward and sawed asunder to give room, one having been removed.

"Years afterwards pyorrhœa began to manifest itself, causing much suffering, and, one by one, loss of teeth. Dentists of Michigan, Missouri, Kansas, and New Mexico treated the mouth, but deemed it sufficient to clean the teeth thoroughly beneath the gums. Finally Dr. Olney, of New Mexico, decided to remove the teeth that were beyond hope, and try the potash remedy. After thoroughly cleansing the teeth, strings were wet in liquor potassæ, wrapped around the teeth, one by one, and thrust beneath the gum as far as possible, being left *in situ* until the gum became purple and the thin edges dropped away. For a time this allayed the pyorrhœa, but only for a time, while the retracted gum exposed the dentine, and the irritation resulting caused extreme suffering. I could eat nothing sweet or sour, hot or cold, and even the cold air of winter was painful, forcing me to obey the wise injunction to breathe through the nose and keep the mouth shut. Fruit I could not eat. My love for apples and oranges, etc., was lost in the suffering they caused. All the comfort I got was in Shakespeare's picture of old age *sans* teeth, and I longed to put off the evil day, for the suffering made me dread the cold forceps; in fact, the dentist kindly warmed them, that their touch might not be worse than the extraction. I finally fell into Dr. E. A. Stebbins's chair, under his hands. The pyorrhœa is gone, through his injection of bichloride of mercury solution into the pockets formed, and by bi-weekly cleansing with the same. The sensitive condition he has largely removed by the application of nitrate of silver. I now eat anything, and can drink cold water without the pleasure of life being destroyed by the acute suffering.

"Very truly,

"W. H. ASHLEY."

This patient was treated in the summer or early fall of 1889. The teeth that were loose are quite firm; the gum is in good condition; there are but *very* few sensitive places on the cementum. After treating for some time with bichloride of mercury (1 to 1000 parts) without satisfactory results, I applied the salts of nitrate of silver to all exposed surfaces of the cementum. The effect was very pleasing to both of us, as his communication indicates. Since then it has been necessary to retouch some places where it has worn off. This patient would have been here to-day if he could have left his family.

Case IV.—A man about seventy years old. Teeth loose, gums receded, considerable pyorrhœa, tartar quickly redepositing after having been removed. After cleaning the teeth thoroughly, in January, 1888, I applied the salts freely to all exposed portions of the cementum. In a few days there was a very marked improvement of the gum. Since that time the teeth have been much more firm, the gums continue in good condition, and the deposit of calculus is *very* much less. I invited this patient to be present, but, being a farmer, he did not like to leave home.

Case V.—In April, 1886, I treated four approximal cavities in the inferior incisors of a young lady. In the fall of 1888, on examination, I found them perfectly secure, and did not disturb them. This spring (so she writes me) she had some filling done by a dentist in Connecticut, who told her that the lower incisor ought to be filled. She endeavored to explain to him that they had been treated, by some means, to arrest the decay, but could not make him take in the situation, and so he filled the cavities, saying that they looked as if they had been filled with amalgam, and that it was all, or nearly all, out. She is confident that they had not decayed since my treatment.

To submit this remedy to a severe test, in February, 1888, I invited the public primary school-children to come to my office to have their decayed teeth treated. Thirty-five of them came. I simply treated them without regard to size of cavities, or dead or live pulps,—not being particular even about removing *débris* from the cavities.

In March, 1889, I called them again for examination and further treatment, if necessary. Not all of them returned. On account of sickness in my family, I did not invite them in 1890.

Within the last few weeks I have called and examined as many as I could get of them. I kept a record of each cavity treated, and will give a summary of results.

I requested each one to let me have the teeth which had been treated whenever they should come out (most of them being temporary teeth), but very few recollected my request. Therefore the specimens are not so numerous as I hoped they would be.

At the examination in 1889 I found not a few good-sized cavities where there was no decay in 1888, and the cavities that were treated in 1888, in the same mouths, were as good as when treated. An illustration of this fact you may see by examining specimen cuspid "Nos. 1 and 2." In that case there was no decay on the labial surface, or where the large approximal cavity is, when the small approximal cavity on the other side of the tooth was treated. I have seen many such cases. Of course many of the teeth treated are gone and their places filled by permanent ones.

These experiments give the following results: Sixty-four cavities, after a little more than one year, show thirty-seven to be successful, fourteen partially successful, thirteen unsuccessful. Twenty-seven cavities, after more than two years, show ten to be successful, five partially successful, twelve unsuccessful. One hundred and forty-two cavities, after more than three years, show eighty-seven to be successful, thirty-three partially successful, twenty-two unsuccessful.

"Successful" are those that seem to have been kept from further decay. "Partially successful" are those where decay has begun around the original cavity, or decay approached from another place in the tooth,—*e.g.*, where a cavity had been treated in the coronal surface and decay had reached it from an approximal surface. "Unsuccessful" means those where all traces of the silver have disappeared.

Many of the unsuccessful cases have been in mouths of patients of delicate constitution,—just the types of those that must visit the dentist *very often* to have teeth filled.

### MANNER OF APPLICATION.

Make, of hard wood, fine, slender points that will enter very small cavities.

Put these points into handles on different angles suitable to reach all portions of the teeth (two points, one on an acute and one on an obtuse angle, will be sufficient).

Pulverize the crystals (owing to impurities in the common lunar caustic sticks, it is much preferable to use the crystals).

The salts are dissolved in an equal amount of water, therefore there should be but little moisture in the cavity, or on the surface to be treated.

Moisten the wood-point a very little, so the powder will stick to it, and then take up on it an amount about the size of the head of a common pin, or more, according to the size of the cavity or sur-

Wood-point set in socket, the apex of which may be round, square, or wedge-shaped, tc suit the operator.

face, and apply to every part of the diseased portion. Apply enough salts and moisture to be sure the whole surface is touched. The salts will take effect in a minute or so.

Waste amalgam scraps rubbed over the treated surface, or cavity, will take up the liberated nitric acid and turn the decay dark instantly. (Since writing this paper I have used silver filings.)

I have not sufficient data to determine whether the application of the amalgam is beneficial or not, but theoretically I think it is.

Silver, instead of wood, points may be used.

Of course the mouth of the patient should be protected during the operation.

Any slight touch to the tongue or other parts of the mouth will do no harm. I never heard a complaint of bad after-results. Some dislike the taste.

Use colored napkins so the stains will not show.

Do not allow the patient to wipe the mouth immediately with a handkerchief for fear of getting it stained.

After the salts have taken effect, and you are through with the treatment, at once inject a copious amount of water to carry away the surplus; also allow the patient to rinse the mouth well.

The *manner* of protecting the patient's mouth from being touched with the salts can be determined readily by each operator. Caution and experience will enable any one to protect the patient's mouth and his own fingers.

SOME POINTS.

Nitrate of silver forms with albumen and fibrin definite compounds, that are insoluble except with a few substances.

It is superficial in its action.

With the phosphates it forms nitrate of lime and phosphate of silver.

With the carbonates it forms carbonate of silver and nitrate of lime.

Superficial decay in labial and buccal surfaces show most favorable results.

Small cavities are more favorable than large ones.

If decay has reached the pulp it is not safe to apply the silver.

In my experiments thus far I have not removed any decay before applying the salts.

Where the gum has receded, and the exposed cementum is sensitive, the effect is very beneficial. In such cases it seems to stimulate the gum to more healthy action.

The liberated nitric acid should be removed.

When asked by patients what the treatment does, I often tell them it kills, embalms, and buries the microbes right in the place where it finds them.

The following patients will now be presented:

CASE I.—Girl. The little pits in buccal surfaces of second lower temporary molars were treated in March, 1886. The apex cavities in upper temporary molars treated in September, 1890. None of these cavities seem to have decayed since treatment.

CASE II.—Boy. The buccal surfaces of temporary molars were treated March, 1886, and have not decayed since. The apices in lower temporary molars were treated in June, 1887, and, having begun to decay again, were re-treated in November, 1889, since which time they seem to have kept well.

CASE III.—Girl. This patient had some cavities treated in 1888 and 1889, which you will observe are in a good state of preservation; while surfaces that appeared sound when the others were treated have large cavities now. For illustration of this case see "cut" of temporary cuspid.

CASE IV.—Girl. This patient had several cavities treated in 1888. A year later nearly all traces of the silver had disappeared, and decay was active. They were then re-treated, and also some new cavities. Two years later all traces of the treatment had disappeared. Please observe that this girl is very nervous and of slight figure,—just the type of patients that often return to us for filling and refilling.

CASE V.—Man. This patient had six small cavities treated in apex surfaces of lower incisors in January, 1886, and have not been touched since. Please observe that the characteristic results of the treatment are perfect, and the decay has been entirely arrested.

CASE VI.—Young lady. In 1888, when fourteen years old, she had nineteen cavities treated in the upper teeth,—most of them very large,—some adjoining fillings. Her health and nervous conditions were such that she could not have fillings put in, and must have relief in some way or lose her teeth. Present condition: first molars decayed all away. Three cavities that were treated have since been filled. The remaining cavities have the characteristics of the treatment, and seem not to be decaying.

## Figures and Tables

**Figs. 1 and 2 f1:**
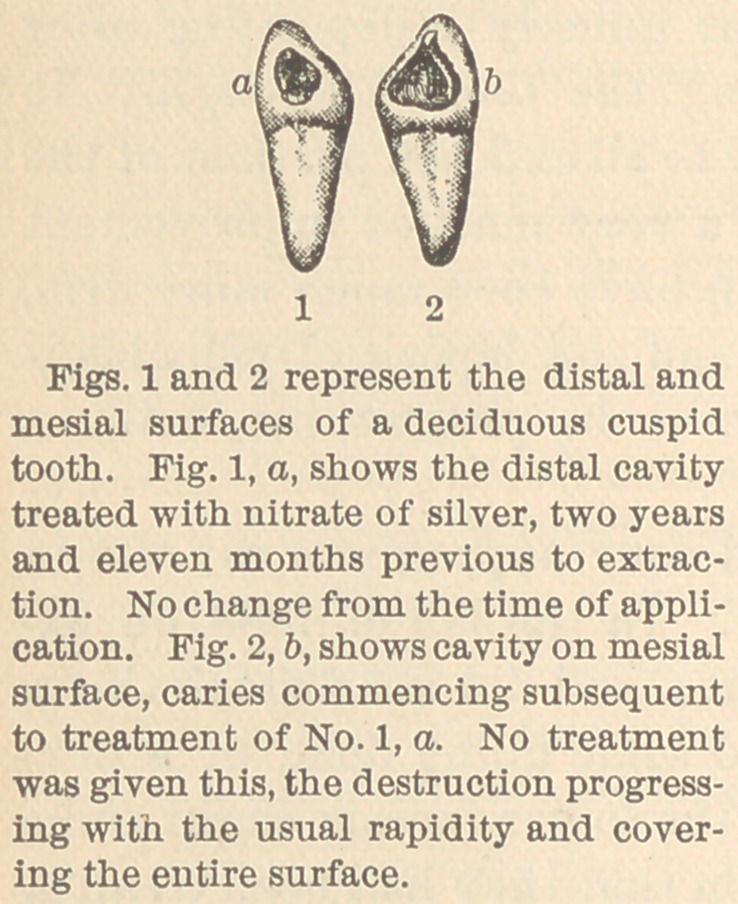


**Figure f2:**